# Implementation of pharmacist recommendations following medication reviews in aged care

**DOI:** 10.3389/fphar.2025.1690600

**Published:** 2025-10-29

**Authors:** Noah C. Ramsey, Gregory M. Peterson, Corinne Mirkazemi, Mohammed S. Salahudeen

**Affiliations:** School of Pharmacy and Pharmacology, College of Health and Medicine, University of Tasmania, Hobart, TAS, Australia

**Keywords:** aged care, medication review, deprescribing, implementation, medication safety

## Abstract

**Background:**

Residential medication management reviews (RMMRs) are a government-funded program in Australia, designed to optimise medication use and enhance safety in aged care residents through reviews conducted by credentialed pharmacists. However, variability in general practitioners’ (GPs) implementation of pharmacists’ recommendations may limit their effectiveness. This study investigated the recommendations made by pharmacists during RMMRs, and the aspects of the recommendations that were associated with their subsequent implementation by GPs.

**Methods:**

This retrospective study analysed RMMRs from 54 aged care facilities across Tasmania, Australia between January 2020 and December 2023. Residents with at least two RMMRs spaced approximately 12 months apart were included, with data extracted from the earliest eligible RMMR as the index. Data sources included medication profiles, residents’ clinical histories, RMMR reports, GP feedback forms, and follow-up RMMRs.

**Results:**

Of the 1646 index RMMRs analysed, 3774 recommendations were made (median: 2 per RMMR), with 50% (n = 1872) fully implemented by the next RMMR 12 months later. Most recommendations (91%, n = 3380) involved a change in therapy, primarily medicine cessation (49%, n = 1810) or dose reduction (21%, n = 799), with implementation rates of 51% and 49%, respectively. Common deprescribing targets included colecalciferol (n = 318, 37% implemented), proton pump inhibitors (n = 123, 43% implemented), statins (n = 145, 48% implemented), and low-dose aspirin (n = 107, 63% implemented). One in six recommendations involved a potentially inappropriate medicine (PIM) according to an Australian resource, such as antipsychotics, benzodiazepines, and opioids. Implementation rates were comparable between deprescribing of PIMs and non-PIMs (51% vs. 50%, p > 0.9). Monitoring-related recommendations were significantly more likely to be implemented than those involving a change in therapy (73% vs. 48%, p < 0.001).

**Conclusion:**

Half of all recommendations were implemented by GPs within 12 months, with most targeting the deprescribing of preventive or high-risk medicines. Future research should identify which recommendations GPs prioritise and the factors influencing their implementation of pharmacists’ medication review recommendations, in order to enhance the effectiveness of RMMRs in aged care.

## 1 Introduction

Inappropriate medicine use in aged care is a growing concern globally and has significant implications for resident safety and healthcare costs (Donaldson et al., 2017). Internationally, over two-thirds of aged care residents are prescribed potentially inappropriate medicines (PIMs), based on tools such as the STOPP criteria (O'Mahony et al., 2023), with prevalence rates as high as 88% (Díaz Planelles et al., 2023). In Australia, medication safety and quality use of medicines is recognised as a national health priority area (Australian Commission on Safety and Quality in Health Care, 2020); however, suboptimal medicine use is a persistent issue in aged care residents, as highlighted by the Royal Commission into Aged Care Quality and Safety in 2021 (Pagone and Briggs, 2021). Up to 75% of all Australian aged care residents are prescribed at least one PIM (Sawan et al., 2024), and medication-related adverse effects account for one in five unplanned hospitalisations among older adults (Parameswaran et al., 2017; Kalisch et al., 2012).

The Australian government funds structured medication reviews in aged care residents (residential medication management reviews; RMMRs) performed by credentialled pharmacists. The RMMR service is initiated by general practitioners (GPs), and is a collaborative process between the GP, the credentialled pharmacist, the resident, and residential aged care facility (RACF) staff. One RMMR is funded for aged care residents every year (Australian Government Department of Health and Aged Care, 2025), with funding introduced in 2020 for up to two follow-up reviews conducted within 9 months of the initial RMMR, as recommended by the Royal Commission (Pagone and Briggs, 2021; Australian Government Department of Health, 2025). RMMRs aim to optimise medicine use, reduce harms, and improve health outcomes for residents of aged care facilities. Similar pharmacist-led reviews have also been adopted internationally (Houle et al., 2014) and are considered a key strategy to minimise medication-related harm in aged care (World Health Organization, 2019a).

Robust evidence for mortality benefits or reductions in hospitalisations after pharmacist-led medication reviews is still lacking and/or inconclusive (Huiskes et al., 2017), although a large study recently reported a modest 4% reduction in 12-month mortality following timely RMMRs (Sluggett et al., 2022a). Moreover, RMMRs have also been shown to be beneficial in identifying and reducing drug-related problems (DRPs) (Chen et al., 2019), and improving the appropriateness of prescribing (Koria et al., 2018), including reducing anticholinergic and sedative burden (McLarin et al., 2016; Nishtala et al., 2009), and psychotropic use (McDerby et al., 2020).

However, variability in the implementation of pharmacists’ recommendations by GPs remains a key barrier to RMMR effectiveness. For example, a systematic review found that while RMMRs identified an average of 2.7–3.9 DRPs per resident, GP acceptance and implementation rates ranged from 45% to 84% (Chen et al., 2019). Understanding and addressing the variability in how GPs implement pharmacists’ medication review recommendations is essential to improving the effectiveness of these reviews. This study aimed to characterise the types of recommendations made by accredited pharmacists during RMMRs, and to identify specific aspects of recommendations that were associated with implementation by GPs.

## 2 Methods

### 2.1 Study design, setting and population

This retrospective study analysed RMMR data provided by Consultant Pharmacy Services (CPS), an organisation employing credentialled pharmacists to conduct medication reviews across Tasmania, Australia. The dataset included RMMRs conducted by nine accredited pharmacists (and one supervised intern) across 54 aged care facilities, encompassing all geographic regions of Tasmania. Eligible participants were aged care residents who received at least two RMMRs approximately 12 months apart, between January 2020 and December 2023. For residents with multiple RMMRs meeting the inclusion criteria, the two earliest RMMRs that met the inclusion criteria were included. Data was extracted from the first RMMR (index RMMR), whilst the second RMMR approximately 12 months later was used to determine the implementation of the recommendations from the index RMMR.

### 2.2 Data collection

CPS maintains paper-based records of their RMMRs, consisting of a list of each resident’s medicines, notes on the resident’s clinical history (diagnoses, laboratory and non-laboratory results) and the final RMMR report (containing recommendations) sent to the GP. Each RMMR report also includes a feedback form for the GP to comment on their acceptance of the pharmacist’s recommendations (and to return to CPS). During the index RMMR, the pharmacist flags which recommendations they believe require follow-up, typically those deemed clinically significant or requiring ongoing monitoring, which triggers a prompt for a follow-up review. Where clinically appropriate, follow-up reviews are conducted approximately 3 months after the index RMMR, with a second follow-up review at six to 9 months post index review, if needed, to assess the progress or outcomes of recommendations. Data was retrospectively extracted by one study investigator (NCR) from the CPS-maintained records (patient medication profiles, full RMMR reports, GP feedback forms, and follow-up RMMRs), and stored in Microsoft Access (Microsoft Corporation, Redmond, WA, United States).

The residents’ clinical diagnoses were extracted and classified according to the World Health Organization’s (WHO) International Statistical Classification of Diseases and Related Health Problems, 10th revision (ICD-10) (World Health Organization, 2019b). Comorbidities were reported using the Charlson Comorbidity Index (CCI) (Charlson et al., 1987), with ICD-10 codes mapped to the CCI categories using the algorithm developed by Quan et al. (2005). The residents’ medication list and medicines involved in the recommendations were documented using the WHO’s Anatomical, Therapeutic and Chemical (ATC) classification system (WHO Collaborating Centre for Drug Statistics Methodology, 2024). PIMs were categorised according to an Australian PIM list for older adults, developed through expert consensus (Wang et al., 2024). Each pharmacist recommendation was categorised according to the reason for the recommendation (e.g., DRP identified) and the type of recommendation made (e.g., medication cessation) according to the DOCUMENT classification system (Williams et al., 2011).

### 2.3 Outcomes and statistical analysis

The acceptance of each recommendation was determined by the GP’s comment on the feedback form, whilst the descriptive implementation rate was determined by comparing index medication lists with those collected at follow-up reviews (completed at 3 months, and 6–9 months), and at the time of the next full RMMR approximately 12 months after the index one. Where available, the reason for the GP’s non-acceptance of the recommendation was categorised according to a list created by the study authors based on a review of the literature (Supplementary Table S1).

Statistical analyses were conducted using R software (RStudio, Boston, MA, United States). For the purpose of analysis, recommendations that were only partially implemented (where the change made by the GP differed from the pharmacist’s recommendation, e.g., a dose decrease when medication cessation was recommended) were classified as not implemented. The implementation of the recommendation at the time of each resident’s full second RMMR (12 months after the index RMMR) was used for all statistical analyses. When acceptance and/or implementation data were unavailable, rates were calculated excluding the missing data. Data was summarised using means and standard deviations for continuous variables with an approximately normal distribution, and medians with interquartile ranges (IQR) for other continuous variables. Normality was visually assessed using histograms. Associations between categorical variables were assessed using Pearson’s chi-square test, while the Mann-Whitney U test was used to compare non-normally distributed continuous variables. A p-value of <0.05 was considered statistically significant.

### 2.4 Ethics approval

This study was approved by the Tasmania Health and Medical Research Ethics Committee (H26567).

## 3 Results

### 3.1 General overview

A total of 1646 index RMMRs conducted between January 2020 and December 2022 met the inclusion criteria (Table 1). The median age of the residents receiving the RMMRs was 84 years (IQR 77–89 years), and 67% were female. Residents had a median of nine recorded diagnoses, and a median unadjusted CCI score of two. The most prevalent diagnoses were hypertension (n = 957, 58%), arthritis (n = 868, 53%), dementia (n = 639, 39%), and depression (n = 606, 37%). Residents were prescribed a median of eight regular medicines, with 58% (n = 956) prescribed at least one regular PIM. Most RMMRs (88%, n = 1456) contained at least one recommendation, with a median of two recommendations per RMMR (IQR 1–3).

**TABLE 1:** Summary of resident and RMMR characteristics.

Characteristic	Number of recommendations on RMMR			
	Overall N = 1,646	≤2 N = 991	≥3 N = 655	p-value
Age (years)	84 (77, 89)	85 (77, 90)	83 (77, 89)	0.029
Sex				0.2
Female	1,102 (67%)	675 (68%)	427 (65%)	
Male	544 (33%)	316 (32%)	228 (35%)	
Number of diagnoses (ICD)	9 (6, 11)	8 (6, 11)	10 (7, 12)	<0.001
CCI score	2 (1, 2)	1 (1, 2)	2 (1, 3)	<0.001
Number of regular medicines	8 (5, 11)	6 (4, 9)	10 (7, 13)	<0.001
Number of as required medicines	3 (2, 5)	3 (1, 5)	3 (2, 5)	<0.001
Polypharmacy (≥5 regular medicines)	1,371 (83%)	738 (74%)	633 (97%)	<0.001
Hyperpolypharmacy (≥10 regular medicines)	671 (41%)	278 (28%)	393 (60%)	<0.001
PIM prescribed (in regular medicines)	956 (58%)	504 (51%)	452 (69%)	<0.001
Year of index RMMR				<0.001
2020	1,040 (63%)	667 (67%)	373 (57%)	
2021	262 (16%)	146 (15%)	116 (18%)	
2022	344 (21%)	178 (18%)	166 (25%)	
Number of recommendations on index RMMR	2 (1, 3)	1 (1, 2)	4 (3, 4)	<0.001
Feedback form completed by GP	800 (49%)	482 (49%)	318 (49%)	>0.9
Follow-up review received at 3 months post index RMMR	979 (59%)	466 (47%)	513 (78%)	<0.001
Second follow-up review received at 6–9 months post index RMMR	401 (24%)	166 (17%)	235 (36%)	<0.001

Median (Q1, Q3); n (%)

Mann-Whitney U test; Pearson’s Chi-squared test.

RMMR, residential medication management review; ICD, international classification of diseases; CCI, charlson comorbidity index; PIM, potentially inappropriate medicine; GP, general practitioner.

Recommendations for PIMs were more often flagged by pharmacists for follow-up than those that were not regarding PIMs (89% vs. 59%, p < 0.001). A follow-up review, initiated based on the flagged recommendations, was conducted approximately 3 months after the index RMMR in 59% (n = 979) of RMMRs, with 24% (n = 401) receiving a second follow-up review six to 9 months after the index RMMR.

### 3.2 Acceptance and implementation of recommendations by GPs

Pharmacists made a total of 3774 recommendations during index RMMRs, of which 99% (n = 3727) had implementation data at the time of the next RMMR 12 months later. Overall, 46% (n = 1747) of recommendations received a response from the GP via the feedback form, with 59% (n = 1036) of those accepted, 14% (n = 238) partially accepted, and 21% (n = 367) not accepted. The GPs’ level of acceptance of the recommendation based on their response on the feedback form was unclear for 106 (6%) recommendations, which included responses such as ‘noted’. The reason for non-acceptance and partial acceptance of the recommendations was documented by GPs for 212 (58%) and 133 (56%) recommendations, respectively. Most of the partially accepted recommendations (80%, n = 106) were due to further follow-up being required before the recommendation could be implemented. Frequently cited reasons for non-acceptance included the prescribers clinical judgement (n = 54, 25%); refusal by the resident or their family (n = 45, 21%), particularly for deprescribing vitamins and benzodiazepines; the recommendation not being considered appropriate (n = 39, 18%), such as for recommendations to deprescribe colecalciferol and proton pump inhibitors; and the medication being managed by another prescriber (n = 21, 10%), mainly for anticholinesterase inhibitors managed by geriatricians.

**FIGURE 1:**
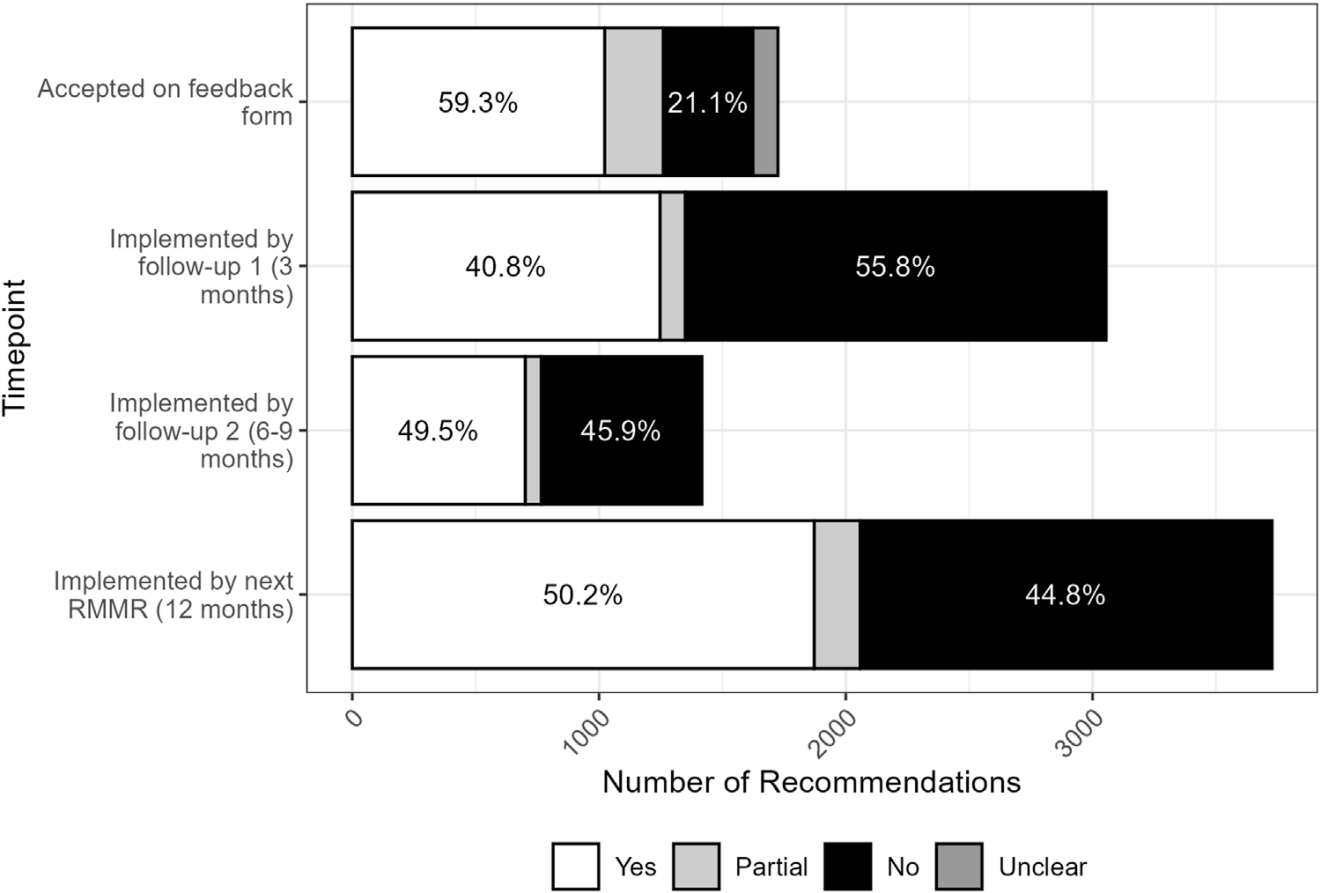
Acceptance and implementation of pharmacist RMMR recommendations by GPs over time (n = 1747 at time of feedback, n = 3066 at follow-up 1, n = 1418 at follow-up 2, n = 3727 at next RMMR).

At the first follow-up review (approximately 3 months post-RMMR), the implementation rate was 41% (1,253/3,066) (Figure 1). This increased to 49% fully implemented (701/1418) by the second follow-up review, conducted six to 9 months after the index RMMR. At the time of the next RMMR (12 months post-index RMMR), 50% (1872/3727) of recommendations had been fully implemented, 5% were partially implemented, and 45% remained unimplemented. Only 4% (n = 76) of recommendations implemented at either the first or second follow-up review were reversed by the time of the next RMMR.

Recommendations for which GPs completed the feedback form had significantly higher 12-month implementation rates (56% vs. 45%, p < 0.001). The completion of a single follow-up review by the pharmacist had no impact on 12-month implementation rates (50% vs. 51%, p = 0.6), however when two follow-up reviews were conducted there was a slight increase in 12-month implementation rates (53% vs. 49%, p = 0.034).

### 3.3 Implementation of recommendations for a change in therapy

**FIGURE 2:**
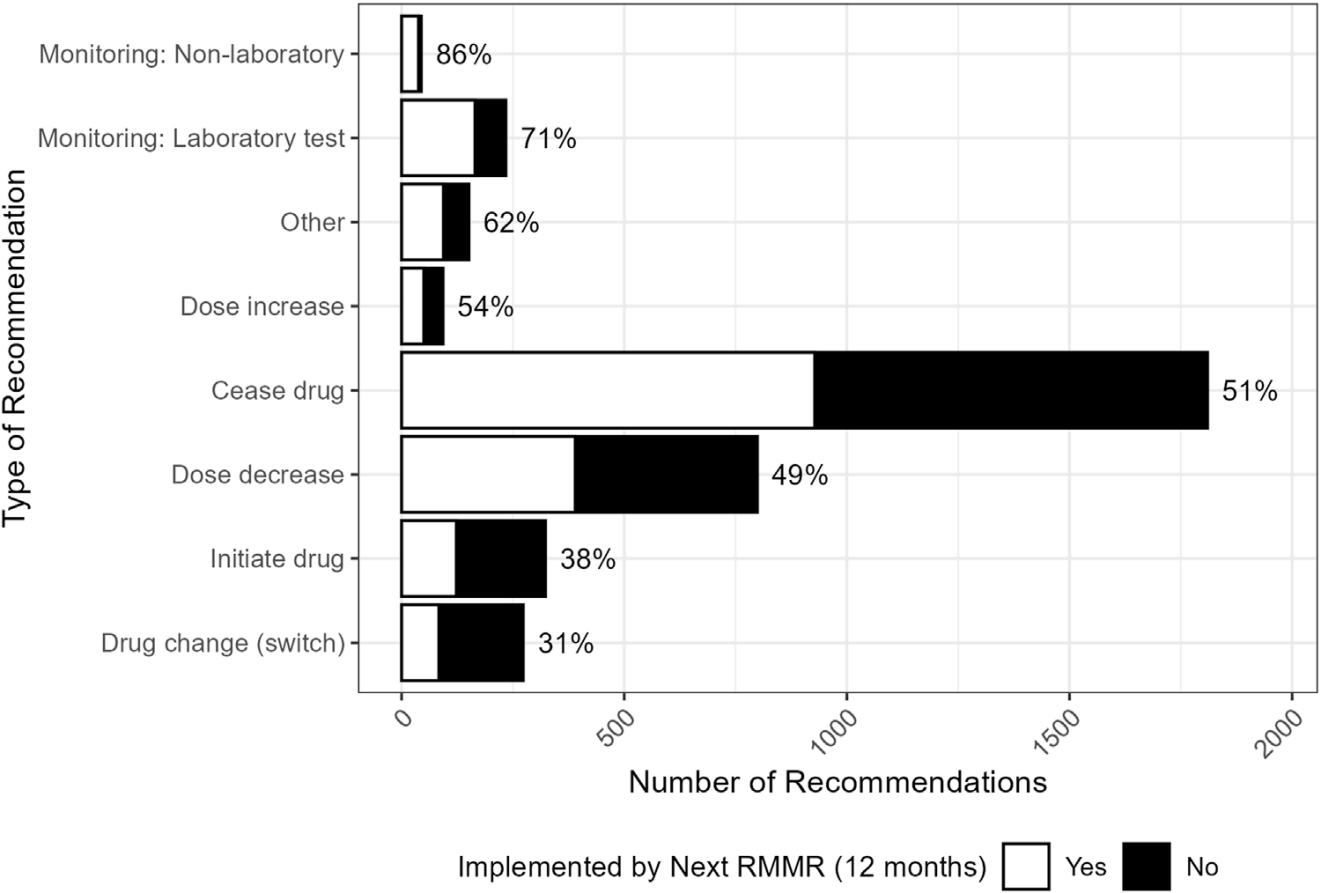
Distribution (and GP implementation rates) by the type of recommendation made by the pharmacist (n = 3727). “Other” included recommendations for: changes to dose frequency and schedule (n = 39), drug brand changes (n = 19), drug formulation changes (n = 16), referrals (n = 13), recommendations relating to the provision of information (n = 13), and any other recommendation that was unable to be classified (n = 43).

**FIGURE 3:**
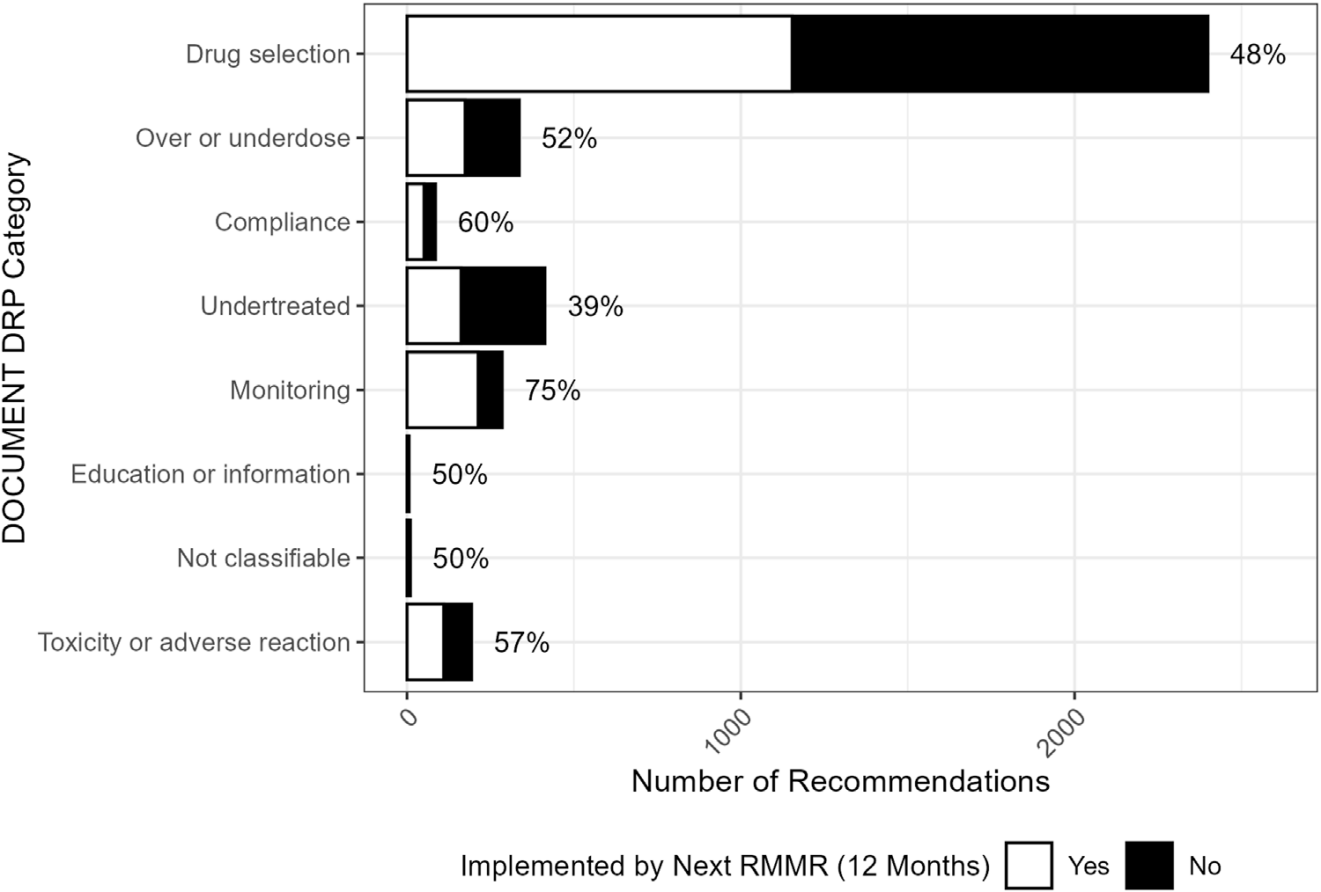
Distribution (and associated GP implementation rates) of index RMMR recommendations across DOCUMENT drug-related problem categories (n = 3727).

The implementation of recommendations at 12 months post-index RMMR, by the type of recommendation, is shown in Figure 2. Almost all recommendations (91%, n = 3380) involved a change in therapy. These were most often for deprescribing recommendations for medication cessation (49%, n = 1810) or dose reduction (21%, n = 799), with implementation rates of 51% and 49%, respectively. These recommendations were commonly associated with drug selection problems and dosing issues categorised with the DOCUMENT system (Figure 3).

Deprescribing recommendations were often made for medicines used without an appropriate indication, including colecalciferol prescribed without calcium supplementation (n = 268, 32% implemented), as well as low-dose aspirin (n = 84, 62% implemented) and statins (n = 73, 55% implemented) prescribed for the primary prevention of cardiovascular disease. Recommendations for antipsychotics prescribed without a clear indication, such as for residents without a history of bipolar disorder or psychotic illness, had a higher implementation rate (n = 13, 77%). Dosing issues commonly involved the prescribed dose being too high (n = 229), particularly paracetamol in low-weight elderly residents (n = 65, 52% implemented).

**FIGURE 4:**
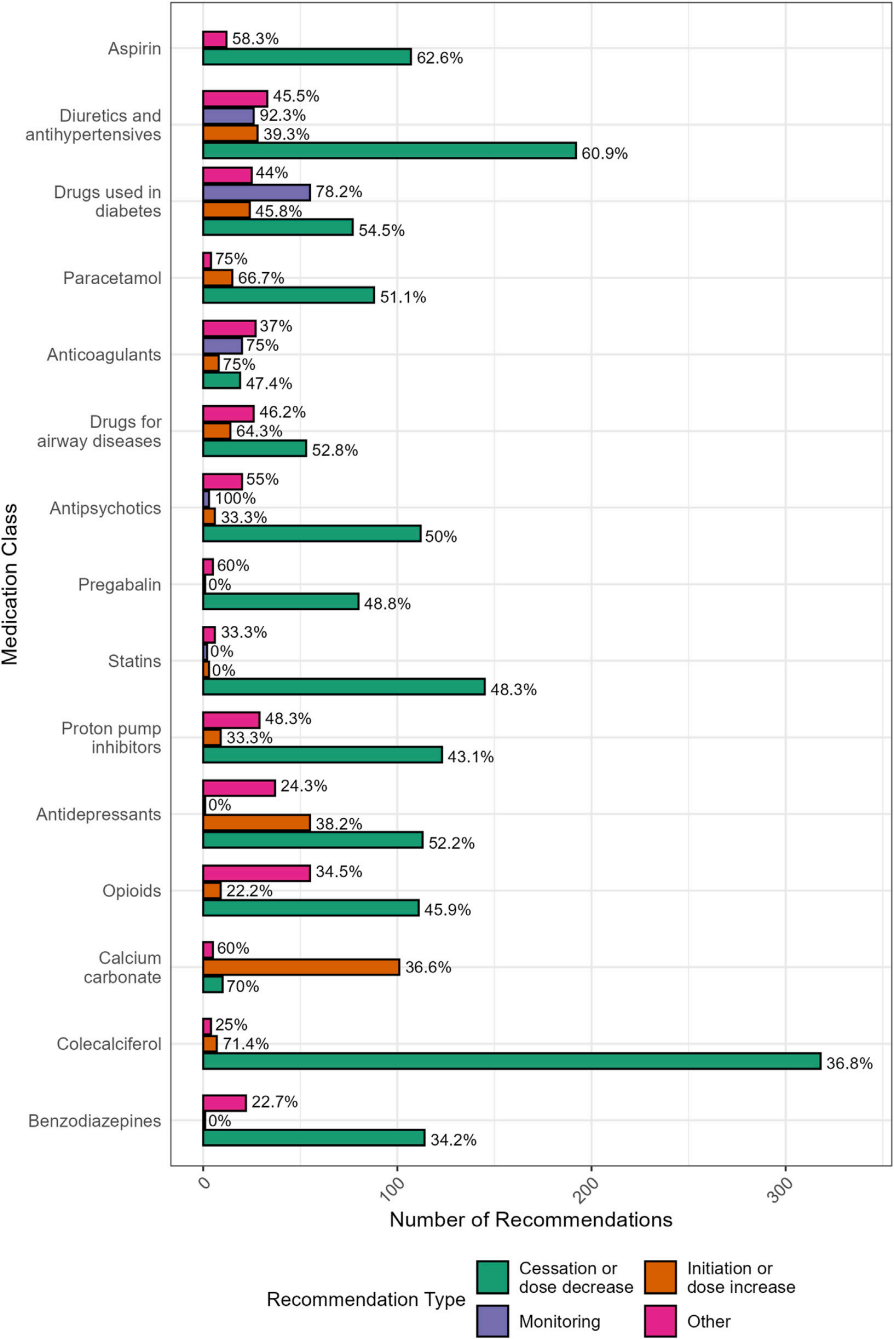
Distribution (and GP implementation rates) of pharmacist recommendations by the medication involved and the type of recommendation (N = 2360). “Other” included recommendations for: drug change (switch) (n = 273), changes to dose frequency and schedule (n = 39), drug brand changes (n = 19), drug formulation changes (n = 16), referrals (n = 13), recommendations relating to the provision of information (n = 13), and any other recommendation that was unable to be classified (n = 43).

The distribution of pharmacist recommendations by the medication involved and the type of recommendation is shown in Figure 4. Recommendations to initiate new medicines had lower implementation rates (38%) and were typically made in response to undertreated conditions. Nearly one-third (31%, n = 101) of these recommendations involved the initiation of preventative calcium, particularly in residents prescribed denosumab, with an implementation rate of 36%.

Recommendations to switch to an alternative medication had the lowest implementation rate overall (31%). These recommendations commonly included opioid rotation to reduce tolerance or switching to a different analgesic (n = 47, 30% implemented) and changing from high-potency to low-potency proton pump inhibitors (PPIs) to facilitate deprescribing (n = 28, 50% implemented).

### 3.4 Implementation of monitoring recommendations

Monitoring recommendations had the highest implementation rates overall (73%). Non-laboratory monitoring, such as blood pressure monitoring for residents prescribed antihypertensive medicines, had an 86% implementation rate. Laboratory monitoring recommendations (n = 234, 71% implemented) included HbA1c and/or renal monitoring for metformin (n = 30, 80% implemented), thyroid function tests for levothyroxine (n = 28, 75% implemented), renal function monitoring for anticoagulants (n = 17, 71% implemented) and uric acid levels for residents prescribed allopurinol (n = 23, 52% implemented). Monitoring was often recommended to ensure doses and ongoing use remained appropriate.

Overall, monitoring recommendations were significantly more likely to be implemented than those involving a change in therapy (73% vs. 48%, p < 0.001). Recommendations citing prior laboratory or clinical values were also implemented at higher rates than those without (59% vs. 49%, p < 0.001).

### 3.5 Implementation of recommendations relating to PIMs and adverse drug reactions

Approximately one in six recommendations (17%, n = 638) made by the pharmacist involved a PIM, with most of these recommendations for deprescribing (72%, n = 461), particularly for antipsychotics (n = 112, 50% implemented), opioids (n = 111, 46% implemented) and benzodiazepines (n = 114, 34% implemented). There was no significant difference in the implementation rates for deprescribing PIM and non-PIM medicines (51% vs. 50%, p > 0.9).

Recommendations for PIMs were more frequently due to an actual adverse drug reaction (ADR) compared to non-PIMs (8.5% vs. 4.5%, p < 0.001). Among ADR-related recommendations, those involving PIMs had a higher implementation rate than those not involving PIMs, however this difference was not statistically significant (65% vs. 54%, p = 0.2). Common ADRs included peripheral oedema and falls due to dihydropyridine calcium channel blockers and nitrates; dry mouth, constipation and weight gain due to antidepressants (particularly amitriptyline, duloxetine, and mirtazapine); and constipation, drowsiness and confusion due to opioids and pregabalin. Antipsychotics were also the source of ADRs, causing drowsiness, oedema and tremors.

## 4 Discussion

Implementation rates for pharmacists’ RMMR recommendations increased from 41% at 3 months post-index RMMR to 50% at the time of the next full RMMR 12 months later. These rates were slightly lower than reported in previous research, including our systematic review, which reported acceptance and implementation rates of pharmacist aged care recommendations ranging from 54% to 79%, with a weighted average of 67% (Ramsey et al., 2025). Similarly, an earlier review focusing on Australian RMMRs reported acceptance rates between 45% and 84% and implementation rates of 58%–72% (Chen et al., 2019). In those two reviews, monitoring recommendations comprised 20% and 27% of all recommendations, respectively, and were associated with higher acceptance and implementation rates than recommendations involving changes to therapy. Consistent with these findings, monitoring recommendations in our study achieved significantly higher implementation rates than therapy changes (73% vs. 48%, p < 0.001), although they comprised less than 10% of all recommendations, likely contributing to the lower overall uptake.

Most recommendations involved deprescribing (either cessation or dose reduction), reaffirming the central role of pharmacists in optimising medicine use in aged care residents (Koria et al., 2018; Sluggett et al., 2022b). While implementation rates for deprescribing (51% for cessation; 49% for dose reduction) were lower than for monitoring, these recommendations are often of greater clinical significance, particularly in reducing medication-related harm. Patient-specific deprescribing interventions have been shown to significantly reduce mortality rates in older adults according to one systematic review (Page et al., 2016). The high rates of polypharmacy observed in this study (83% of residents, with 41% taking 10 or more medicines), together with its widespread prevalence in aged care (Page et al., 2023) and links to increased hospitalisation (Davies et al., 2020) and mortality (Chang et al., 2020), underscore the importance of deprescribing. Conversely, concerns about polypharmacy and medication burden may have contributed to the lower implementation rates for recommendations to initiate new medicines (38%) and address undertreated conditions (39%). In addition, research has shown that older adults are generally more receptive to stopping medicines than starting new ones, with nearly 80% willing to deprescribe one or more medications if recommended by their doctor, compared to just over half who were willing to take more medications for their health conditions (Kalogianis et al., 2016). Notably, resident/family refusal accounted for 21% of documented non-acceptances in this study.

More than half (58%) of the included residents were prescribed a regular PIM, slightly lower than PIM rates reported in previous studies of Australian aged care residents (Sawan et al., 2024; Haider et al., 2022). These studies reported PIMs using the Beers criteria (Panel, 2023), which may disclose more PIMs than the Australian-specific resource used for this study. Approximately one in six recommendations involved a PIM, most of which were for deprescribing. Two-thirds of these recommendations targeted antipsychotics, benzodiazepines, and opioids. This is particularly important given the high rates of inappropriate prescribing of these medicines in aged care (Westbury et al., 2013), and their contribution to ADRs and hospitalisations (Kalisch et al., 2021). In this study, ADRs were more frequently cited as the reason for PIM-related recommendations compared to non-PIMs. Although implementation rates were higher for ADR-related PIM recommendations (65% vs. 54%), the difference was not statistically significant. Overall implementation rates were also similar for deprescribing recommendations for PIM and non-PIM medicines. Notably, recommendations for antipsychotic medicines used without a clear indication had high implementation rates (77%), which is encouraging given the Aged Care Royal Commission identified the inappropriate use of antipsychotics as a form of chemical restraint as a significant issue (Pagone and Briggs, 2021). In contrast, benzodiazepine (34%) and opioid (46%) deprescribing were less frequently implemented, likely reflecting concerns about adverse drug withdrawal effects or symptom control (Turner et al., 2016; Djatche et al., 2018). Studies have shown that the deprescribing of these medicines can be challenging due to patient reluctance (Kelley et al., 2023), the drawn out withdrawal process (Niznik et al., 2022), and a lack of resources to address the underlying issues (Niznik et al., 2022). Australia has a high prescribing rate of opioids (Ju et al., 2022), and while there have been changes to increase restrictions on prescribing, the hospitalisation rates associated with opioids have remained unchanged (Nielsen et al., 2025), highlighting the need to increase the implementation of these deprescribing recommendations.

Implementation patterns also varied by medication class and appeared to be influenced by both the strength of supporting evidence and the perceived clinical relevance of the recommendation. For example, aspirin deprescribing achieved relatively high uptake (63%), supported by updated guidelines and the ASPREE trial’s 2018 findings of increased bleeding risk in older adults (McNeil et al., 2018). Similarly, recommendations that referenced laboratory or clinical values were more likely to be implemented than those without, which may explain the higher implementation rates observed for medicines used in diabetes, diuretics, and antihypertensives. These medicines were also associated with a higher proportion of monitoring recommendations. On the other hand, the most common recommendation made by pharmacists was for the deprescribing of colecalciferol, which had a low uptake (37%). GPs often judged this recommendation as not clinically appropriate, despite evidence that colecalciferol without calcium co-supplementation has no proven impact on falls, fracture, or bone mineral density (Bolland et al., 2018). Recent updates to Australian guidelines in 2024 (notably, after the study period) recommend the use of calcium and vitamin D for fracture prevention in aged care residents where deficiency is more prevalent (Wong et al., 2025). The reluctance to deprescribe colecalciferol may reflect conflicting evidence and variation in clinical guidelines, in contrast to the clearer consensus regarding the deprescribing of aspirin used for primary prevention of cardiovascular disease. Similarly, recommendations to initiate calcium also had low implementation rates (36%), despite being commonly recommended to manage the risk of hypocalcaemia in residents prescribed denosumab (Denosumab: Product Information, 2025).

This study has several strengths. It includes a large sample of RMMRs conducted by nine pharmacists (and one supervised intern) across 54 of Tasmania’s 67 RACFs, strengthening the generalisability of the results (Australian Institute of Health and Welfare, 2025a). The median age of residents (84 years), and gender distribution (67% females), aligned with national aged care data (median age at admission 85 years; 66% female), reinforcing the relevance of the results to the Australian aged care population (Australian Institute of Health and Welfare, 2025b). Access to written responses from GPs, including reasons for not accepting recommendations, provided valuable insights into their clinical decision making. In addition, implementation data collected across multiple timepoints provided insight into both the timeline of recommendation uptake and the sustainability of implemented pharmacist recommendations (Australian Government Department of Health, 2025).

However, there are some limitations that should be considered. The pharmacists conducting the RMMRs were all employed by the same organisation, and the study only included residents of Tasmanian aged care facilities therefore the recommendations might not be generalisable to those made by other credentialed pharmacists. Most RMMRs were conducted in 2020 during the COVID-19 pandemic, which may have influenced the GPs ability to implement recommendations and access RACFs. Only half of the recommendations had documented prescriber feedback, limiting the acceptance analysis. It is also possible that some recommendations may have been implemented and later reversed before the pharmacist follow-up, which would not have been captured. Additionally, the clinical relevance and appropriateness of the pharmacists’ recommendations was not assessed, and there may have been some recommendations where implementation was not appropriate. External factors known to influence implementation, such as interprofessional relationships, communication, and facility-related were not captured (McDerby et al., 2020; Ramsey et al., 2025; Kwint et al., 2013).

Future research should examine GP, resident, and facility-level factors influencing the uptake of pharmacist recommendations. Identifying which recommendations are prioritised by prescribers, and the factors that influence their decision to implement pharmacists’ recommendations is critical for improving effectiveness. Importantly, recommendations should remain clinically relevant, rather than being tailored towards what is most likely to be accepted. Strategies that improve prescriber confidence, align recommendations with current evidence, and facilitate shared decision-making may enhance implementation and ultimately improve medication safety in aged care. This includes prioritising increased verbal communication and collaboration between pharmacists, GPs, aged care staff and residents to ensure recommendations are relevant and facilitate increased implementation (Ramsey et al., 2025; Kwint et al., 2013).

## 5 Conclusion

This large retrospective study highlights the real-world impact of pharmacist-led RMMRs in aged care settings, with half of all recommendations implemented by GPs within 12 months. Most recommendations targeted deprescribing of preventive and high-risk medicines, highlighting the pharmacist’s role in optimising medication use. Recommendations supported by clinical or laboratory data were more likely to be implemented, while uptake varied by drug class and recommendation type. Lower implementation rates for medicine initiation and certain deprescribing recommendations, particularly for benzodiazepines and opioids, highlight ongoing challenges. Strengthening strategies to improve the uptake of clinically important recommendations including increasing the collaboration between pharmacists, GPs and residents via initiatives such as aged care on-site pharmacists remains critical to enhance the effectiveness of medication reviews and reduce medication-related harm in aged care.

## Data Availability

The datasets presented in this article are not readily available because the data analysed in this study were obtained under agreement from CPS. Only individuals named in the ethics application are authorised to access the data. Requests for data access can be directed to the corresponding authors and will be considered on reasonable grounds in consultation with the data custodian. Requests to access the datasets should be directed to Noah C. Ramsey, noah.ramsey@utas.edu.au.
