# Supplementary material for: Implementation of pharmacist recommendations following medication reviews in aged care
Source: Front Pharmacol. 2025 Oct 29;16:1690600. doi: 10.3389/fphar.2025.1690600 (PMC12605112; doi:10.3389/fphar.2025.1690600)
Supplement: Supplementary file 1 [file Table1.docx]

Supplementary Material

Supplementary Table 1. Reason for the non-acceptance of the pharmacist’s recommendation documented by the GP on the feedback form

| **Reason for non-acceptance** |
| --- |
| Further follow-up required |
| Patient or family will refuse |
| Prescriber's clinical judgement |
| Recommendation not relevant or appropriate |
| Medication from another prescriber |
| Difficult to manage condition |
| Change made prior to recommendation |
| Previously trialled |
| Based on lab values that have likely corrected |
| Other issues more pressing |
| Other |
